# First demonstration of an all-solid-state optical cryocooler

**DOI:** 10.1038/s41377-018-0028-7

**Published:** 2018-06-06

**Authors:** Markus P. Hehlen, Junwei Meng, Alexander R. Albrecht, Eric R. Lee, Aram Gragossian, Steven P. Love, Christopher E. Hamilton, Richard I. Epstein, Mansoor Sheik-Bahae

**Affiliations:** 1 Los Alamos National Laboratory, P.O. Box 1663, Los Alamos, NM 87545 USA; 2 Department of Physics & Astronomy, University of New Mexico, Albuquerque, NM 87131 USA

## Abstract

Solid-state optical refrigeration uses anti-Stokes fluorescence to cool macroscopic objects to cryogenic temperatures without vibrations. Crystals such as Yb^3+^-doped YLiF_4_ (YLF:Yb) have previously been laser-cooled to 91 K. In this study, we show for the first time laser cooling of a payload connected to a cooling crystal. A YLF:Yb crystal was placed inside a Herriott cell and pumped with a 1020-nm laser (47 W) to cool a HgCdTe sensor that is part of a working Fourier Transform Infrared (FTIR) spectrometer to 135 K. This first demonstration of an all-solid-state optical cryocooler was enabled by careful control of the various desired and undesired heat flows. Fluorescence heating of the payload was minimized by using a single-kink YLF thermal link between the YLF:Yb cooling crystal and the copper coldfinger that held the HgCdTe sensor. The adhesive-free bond between YLF and YLF:Yb showed excellent thermal reliability. This laser-cooled assembly was then supported by silica aerogel cylinders inside a vacuum clamshell to minimize undesired conductive and radiative heat loads from the warm surroundings. Our structure can serve as a baseline for future optical cryocooler devices.

## Optical Refrigeration: an all-solid-state cryocooler becomes a reality

The demonstration that an arbitrary load can be successfully cooled by optical refrigeration bodes well for the future for a new breed of solid-state optical cryocoolers. Markus Hehlen and coworkers from Los Alamos National Laboratory and the University of New Mexico built the cooler from a crystal of YLF:Yb that was pumped by a fiber laser. The device cools due to anti-Stokes fluorescence from the crystal, and a HgCdTe sensor was attached to the cooling crystal via a thermal link made from undoped YLF and a copper cold-finger. Upon application of 47W of 1020 nm pump laser light, the optical cryocooler reduced the temperature of the sensor to 135 K from room temperature. The YLF:Yb crystal was placed inside a Herriott cell optical cavity while the entire cooler was placed inside a vacuum chamber in order to control heat flows.

## Introduction

All-optical cooling of a solid was first observed in 1995 by Epstein et al.^1^, and extensive developments over the past two decades in materials, characterization techniques, and optical designs have laid the groundwork for practical applications. Early work focused on the cooling of Yb^3+^-doped fluoride glasses (e.g., ZBLANP:Yb^3+^), the cooling efficiency of which was later found to be limited by the substantial inhomogeneous broadening of the Yb^3+^ crystal-field transition in the amorphous glass host^2^. The much smaller inhomogeneous broadening in Yb^3+^-doped fluoride crystals (e.g., YLiF_4_:Yb^3+^) allowed for higher cooling efficiencies, which helped enable the breakthrough into the cryogenic regime in 2010^3^. This breakthrough has fueled further research into solid-state optical refrigeration^4,5^, which is currently the only technology that can provide truly vibration-free cooling to cryogenic temperatures^6^.

Solid-state laser cooling is achieved using anti-Stokes fluorescence, a process in which the average wavelength of the fluorescence ($\bar \lambda _f$) emitted by a material is shorter than the wavelength (*λ*) of the laser used for excitation^7^. The cooling efficiency *η*_*c*_ is given by the ratio of the cooling power (*P*_cool_) and absorbed power (*P*_abs_):^8^

1 $$\eta _c\,{\mathrm{ = }}\frac{{P_{\mathrm{cool}}}}{{P_{\mathrm{abs}}}} = \eta _{\mathrm{ext}}\eta _{abs}\frac{\lambda }{{\bar \lambda _f}} - 1$$

where *η*_ext_ = *η*_*e*_*W*_*r*_/(*η*_*e*_*W*_*r*_+*W*_*nr*_) is the external quantum efficiency and *η*_abs_ = *α*_*r*_(*λ*)/[*α*_*r*_(*λ*)+*α*_*b*_(*λ*)] is the absorption efficiency. Here, *W*_*r*_ and *W*_*nr*_ are the radiative and non-radiative decay rates of the emitting state, respectively; *η*_*e*_ is the fluorescence escape efficiency; *α*_*r*_(*λ*) is the resonant absorption coefficient of the cooling transition; and *α*_*b*_(*λ*) is the parasitic background absorption coefficient. The radiative process of cooling by anti-Stokes fluorescence competes with a variety of non-radiative processes that reduce *η*_ext_ and *η*_abs_ by multiphonon relaxation of the emitting state (*W*_*nr*_) and absorption by impurities (*α*_*b*_), respectively. Yb^3+^-doped solids have received particular attention because Yb^3+^ can be excited with high-power lasers of ~1 μm wavelength and can emit with high quantum efficiency due to its simple energy-level structure. However, laser cooling of Yb^3+^-doped solids to cryogenic temperatures is only practical if *η*_ext_*η*_abs_ exceeds 0.98–0.99, a challenging requirement that necessitates a cooling crystal with exquisite purity^9^. High-purity YLiF_4_ crystals doped with 10 mol% Yb^3+^ (YLF:10%Yb) have recently been cooled to 91 K by exciting them with a laser tuned to the E4→E5 crystal-field transition of Yb^3+^ at 1020 nm^10^.

Most solid-state laser cooling studies to date have focused on the optical refrigeration of the laser-cooling material itself^4^ or a load that is transparent to fluorescence^11^. For practical applications, however, it is necessary for the laser-cooling material to refrigerate an attached arbitrary external payload, such as a sensor or electronic component. The primary challenges for advancing from a basic laser-cooling setup to a practical optical cryocooler device involve: (1) managing the numerous heat and radiation flows in the system and (2) providing a sturdy, thermally insulating support structure for the laser-cooled assembly. In this article, we address these challenges and describe the design and first experimental demonstration of a solid-state optical cryocooler capable of refrigerating a HgCdTe infrared sensor to 135 K. This device is the only solid-state cooling device that works in the cryogenic regime, i.e., in the temperature range of liquid cryogens, such as Xe, with a boiling point of 166 K. The most common solid-state refrigerators, thermoelectric coolers, cannot achieve temperatures nearly as low as 135 K.

**Fig. 1:**
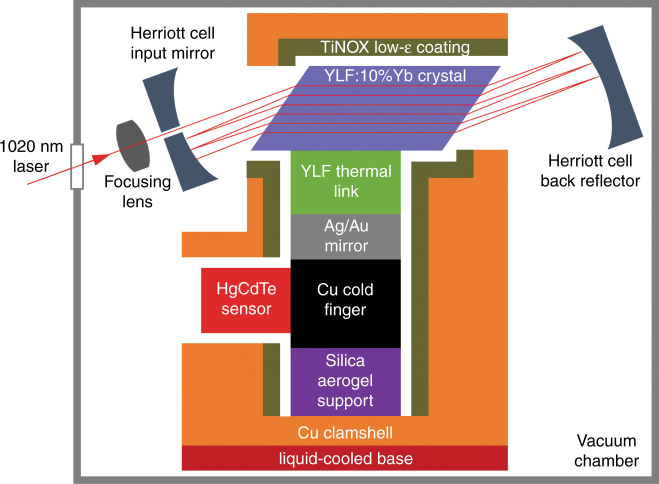
Block diagram (not to scale) showing the components of the solid-state optical cryocooler. The laser-cooling material (blue) is placed inside an astigmatic Herriott cell to enable multi-pass excitation by the pump laser. The sensor payload (red) is connected by a coldfinger (black), mirror (gray), and thermal link (green) to the laser-cooling material. A support element (purple) provides the mounting of this assembly within a closely fitting clamshell (orange), which is lined with a low-emissivity coating (olive) and mounted onto a liquid-cooled base (dark red). The cryocooler is contained within a vacuum chamber (gray)

Figure 1 shows a block diagram of the solid-state optical cryocooler architecture used in this study. The payload is attached via a coldfinger, mirror, and thermal link to the laser-cooling material. This assembly (refrigerator cold side) is mounted with a support element and surrounded by a closely fitting clamshell (refrigerator hot side), which is lined with a low-emissivity coating. The breakthrough performance of the present solid-state optical cryocooler is enabled by the materials and geometries of both the thermal link and support element combined with a high-performance YLF:10%Yb^3+^ crystal. The design of various components and the performance of the optical cryocooler are discussed in detail in the following sections.

## Materials and methods

### Optical components

 We used the YLF:10%Yb laser-cooling crystal that was cooled to 91 K in a previous study^10^. This  crystal was cut so that the linearly polarized pump laser has $\vec E\parallel \vec c$ because the absorption cross section of the E4→E5 crystal-field transition in a YLF:Yb crystal at 1020 nm is ~1.7× greater in this geometry than that with $\vec E \bot \vec c$ polarization^12^.

**Fig. 2:**
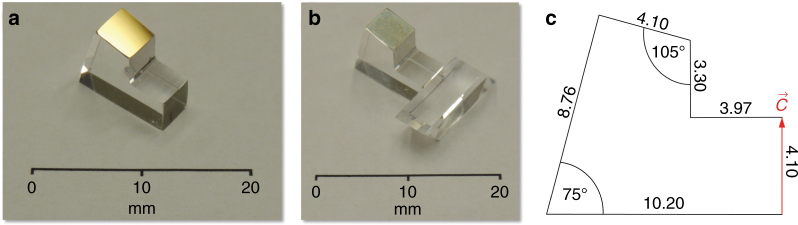
Geometry, fabrication, and bonding of the YLF thermal link. **a** Fabricated YLF thermal link with a sputtered Ag/Au mirror on the cold-finger interface. **b** YLF thermal link AFB-bonded to the Brewster-cut YLF:10%Yb cooling crystal. **c** Cross sectional view and dimensions (in mm) of the δ = 15° geometry with a 90° inside angle. The dimension in the orthogonal direction is 4.10 mm. The orientation of the ${\vec{\mathrm c}}$ axis is indicated, and it is parallel to the ${\vec{\mathrm c}}$ axis of the YLF:10%Yb crystal

The thermal link was fabricated from a Czochralski-grown undoped YLF crystal boule (AC Materials, Tarpon Springs, FL), which was found to have low parasitic background absorption coefficients of $\alpha _b\left( {\vec E\parallel \vec c} \right) = 9.6 \times 10^{ - 5}$ cm^−1^ and $\alpha _b\left( {\vec E \bot \vec c} \right) = 4.4 \times 10^{ - 5}$ cm^−1^, as determined by exposing the sample to a 1020-nm laser (43 W) and measuring the resulting temperature increase relative to a reference heat load using a thermal camera (Nanocore 640, L3 Communications Corporation, Garland, TX, USA). The YLF thermal link was fabricated such that its $\vec c$ axis was aligned parallel to the $\vec c$ axis of the YLF:10%Yb crystal at the respective mating interface (Fig. 1). This crystallographic alignment minimized the thermal stresses induced by the anisotropic coefficients of thermal expansion (CTE) of YLF. The thermal link surface that was attached to the coldfinger was subsequently coated with a metallic mirror by first etching the YLF in an argon plasma followed by sputter deposition of 200 nm of silver, followed by 200 nm of gold. This produced a silver mirror with high reflectance on the inside of the thermal link that was protected from oxidation by the gold layer. Figure 2a, b show the mirrored YLF thermal link before and after Adhesive-Free Bonding^13,14^ (AFB^®^ by Onyx Optics, Inc., Dublin, CA) to the YLF:10%Yb crystal, respectively. The elevated temperatures during the AFB process caused interdiffusion of the Ag and Au layers, as shown by the discoloration of the external gold layer in Figure 2b. No discoloration of the optically important inside Ag mirror was observed. Furthermore, no mechanical failure was observed when temperature cycling an equivalent assembly 22 times from 300 to 75 K (at −6.9 K/min) and back to 300 K (at +21.6 K/min), demonstrating that a thermo-mechanically reliable AFB between undoped YLF and YLF:10%Yb was achieved.

Silica aerogels were synthesized by a sol–gel process, followed by drying in supercritical methanol, which yielded hydrophobic aerogels that reversibly adsorbed <2 wt% water during long-term storage in ambient air^15^. This process also allowed custom aerogel shapes to be directly molded during synthesis. We fabricated hydrophobic silica aerogels with a 0.1 g/cm^3^ density in the shape of 5 mm diameter × 5 mm tall cylinders as the basic support element. The samples were found to absorb <2 × 10^−4^ of the incident power of a 1015-nm laser and were therefore expected to produce a negligible heat load when exposed to YLF:Yb fluorescence.

The solid-state optical cryocooler was placed in a vacuum chamber (5 × 10^−7^ Torr), and the YLF:10%Yb crystal was excited by a linearly-polarized continuous-wave fiber laser (IPG Photonics, Inc., custom-made) that provided 47 W at 1020 nm. The temperature of the coldfinger was monitored with a calibrated silicon diode (LakeShore Cryotronics, DT-670-SD) located in the coldfinger base. In addition, non-contact differential luminescence thermometry (DLT)^3^ was used to monitor the temperature of the cooling crystal by collecting fluorescence with a multimode optical fiber inserted flush with the clamshell wall near the YLF:10%Yb crystal.

### Thermal model

**Fig. 3:**
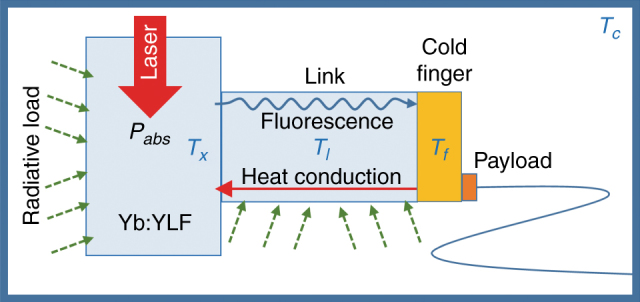
Three-body thermal model of the optical cryocooler. The model consists of a cooling crystal (YLF:Yb) at temperature *T*_*x*_ and a thermal link (YLF) with a mid-length temperature of *T*_*l*_ connected to a copper coldfinger at temperature *T*_*f*_. These elements are placed in a vacuum clamshell whose walls are coated with low-emissivity materials at a constant (ground) temperature of *T*_*c*_

The dynamics of the observed temperature changes of the cooling crystal and coldfinger follow an intricate interplay between the absorbed laser power, cooling efficiency, parasitic losses (due to fluorescence absorption) at interfaces, radiative loads, conductive loads, and useful loads (e.g., heat lift from the sensor). A rigorous model that numerically solves the 3-dimensional heat equation including the exact structure of the optical cooler could, in principle, reproduce the observed thermal dynamics. However, we were able to determine the essential features and underlying parameters by considering a simple 3-element model, as depicted in Figure 3, that consisted of the YLF:Yb cooling crystal (at temperature *T*_*x*_) connected to the coldfinger (at temperature *T*_*f*_) by a thermal link (at temperature *T*_*l*_), which in this case, was also constructed from a YLF (undoped) crystal.

Assuming that the adhesive-free bond between the cooling crystal and thermal link introduces negligible thermal resistance and parasitic loss, the equations governing the temperature evolution of the three elements are approximated as follows:

2 $$C_x\frac{{\mathrm{d}T_x}}{{\mathrm{d}t}}{\mathrm{ = }} - \eta _c\left( {T_x} \right)P_{abs}\left( {T_x} \right) + \epsilon _x\sigma \left( {T_c^4 - T_x^4} \right)A_x \\ + K_l\left( {T_x} \right)\left( {T_l - T_x} \right)$$

3 $$C_l\frac{{\mathrm{d}T_l}}{{\mathrm{d}t}} = - K_l\left( {T_x} \right)\left( {T_l - T_x} \right) + \epsilon _l\sigma \left( {T_c^4 - T_l^4} \right)A_l \\ + K_f\left( {T_l} \right)\left( {T_f - T_l} \right)$$

4 $$C_f\frac{{\mathrm{d}T_f}}{{\mathrm{d}t}}{\mathrm{ = }} - K_f\left( {T_l} \right)\left( {T_f - T_l} \right) + \epsilon _f\sigma \left( {T_c^4 - T_f^4} \right)A_f \\ + \beta _l\eta _{\mathrm{ext}}\left( {T_x} \right)P_{\mathrm{abs}}\left( {T_x} \right) + P_{\mathrm{lift}}$$

where $C_{x,l,f} = V_{x,l,f}C_{x,l,f}^v\left( T \right)$, with *V*_*x*,*l*,*f*_ and $C_{x,l,f}^v$ denoting the known volume and heat capacity (J/K/m^3^), respectively, of the cooling crystal (*x*), link (*l*), and coldfinger (*f*). The temperature dependence of the YLF heat capacity is taken from Ref. 16. *P*_abs_(*T*) =* P*_in_*η*_cpl_ is the absorbed power drawn from the incident power *P*_in_ (at λ = 1020 nm). Here, *η*_cpl_ = 1−exp(−2*N*_rt_*α*_*r*_(*λ*,*T*)*L*_*x*_) is the laser coupling efficiency, where *N*_rt_ is the number of roundtrips the laser makes through the crystal of length *L*_*x*_ placed inside the astigmatic Herriot cell^17^ and *α*_*r*_(*λ*,*T*_*x*_) is the resonant absorption coefficient of the Yb^3+^ crystal-field transition^17^. *K*_*l*_(*T*_*l*_) = 2*κA*/*L*_*l*_ is the effective thermal conductance of the link with a cross-sectional area of *A* and an effective length *L*_*l*_. The factor of 2 arises from the fact that *T*_*l*_ is the temperature of the link halfway between the crystal and the coldfinger. *κ*(*T*_*l*_) is the temperature-dependent thermal conductivity of the thermal link (undoped YLF).^16^ *A*_*x*_, *A*_*l*_, and *A*_*f*_ denote the total surface area of the cooling crystal, link, and cold-finger, respectively. ${\it{\epsilon }}_x \approx {\it{\epsilon }}_l$ are the *effective* emissivities, *ε*/(1 + *χ*), of YLF in a tight clamshell coated with a low emissivity (*ε*_*c*_) TiNOX solar absorber (Almeco GmbH, Bernburg, Germany) held at constant temperature *T*_*c*_, and ${\it{\epsilon }}_f$ is the effective emissivity of the copper coldfinger. Here, *χ*=(1−*ε*_*c*_)*ε*_*l*_*A*_*l*_/*ε*_*c*_*A*_*c*_ (Ref. [11]), where *ε*_*l*_ and *ε*_*c*_ are the emissivities and *σ* is the Stefan-Boltzmann constant. *β*_*l*_ represents the fraction of the total fluorescence power that effectively leaks though the link and is consequently absorbed by the coldfinger. *P*_lift_ is the useful thermal load (payload heat lift) and small parasitic load from the sensor wires and aerogel supports.

## Results and discussion

### Cooling crystal and Herriott cell

As shown in Eq. (1), the cooling power of a solid-state optical refrigerator linearly scales with the laser power absorbed by the cooling crystal (YLF:Yb), *P*_abs_ = *P*_in_*η*_cpl_, which in turn depends on *N*_rt_, *α*_*r*_, and *L*_*x*_. The temperature dependence of *α*_*r*_ scales as *α*_*r*_(*T*_*x*_)∝[1+exp(*δE*_*g*_/*k*_*B*_*T*_*x*_)]^−1^, where *δE*_*g*_ ≈ 460 cm^−1^ is the width of the ^2^F_7/2_ ground-state multiplet in YLF:Yb, and *k*_*B*_ is the Boltzmann constant^17^. The decrease of *α*_*r*_ at low temperatures requires *N*_rt_ to be ~13× greater at 135 K than at 300 K to realize the same *P*_abs_. To realize a large *P*_abs_, the Brewster-cut YLF:10%Yb crystal was placed inside an astigmatic Herriott cell^17^ formed by a concave spherical input mirror (*R* = 50 cm) and a cylindrical back reflector (*R*_*x*_ = ∞, *R*_*y*_ = 50 cm) with the laser focused into the cell with a spherical lens (*f* = 5 cm) through a 400 μm diameter center hole in the input mirror. We estimate *N*_rt_ ≈ 40 in the final configuration, which yields *η*_cpl_ > 0.9999 with *L*_*x*_ = 1.11 cm and *α*_*r*_ = 0.12 cm^−1^ at 1020 nm and 135 K for $\vec E\parallel \vec c$.

### Thermal link

Attaching the payload directly to the YLF:Yb crystal is impractical. Although this would provide an excellent thermal contact, the payload would be directly exposed to YLF:Yb fluorescence and absorb radiation that could exceed *P*_cool_. Therefore, a transparent thermal link was inserted between the YLF:Yb crystal and payload (Fig. 1) to reduce the amount of fluorescence reaching the payload while maintaining an efficient thermal path between the payload and YLF:Yb crystal^18,19^. The thermal, mechanical, and optical properties of the resulting interface between the YLF:Yb crystal and thermal link require careful consideration. The use of adhesives to bond the two elements can introduce optical absorption and corresponding heating. We avoided this by bonding the cooling crystal and thermal link by van-der-Waals forces in an Adhesive-Free Bond (AFB®)^13,14^. Although sapphire is an attractive thermal-link material due to its low optical absorption and high thermal conductivity (*κ* ≈ 400 W/m K at 100 K^20^), our preliminary experiments exploring the AFB of YLF:10%Yb and sapphire found frequent bond failures upon thermal cycling from 300 to 77 K. The stress at the AFB interface induced by a temperature change Δ*T* is proportional to Δ*T*Δ*α*, where Δ*α* is the difference in the coefficients of thermal expansion (CTE) of the two bonded materials^21^. The CTE of sapphire (*α*_*a*_ = 5.22 × 10^−6^ K^−1^ and *α*_*b*_ = 5.92 × 10^−6^ K^−1^ at 296 K^20^) and YLF (*α*_*a*_ = 13.0…14.3 × 10^−6^ K^−1^ and *α*_*c*_ = 8.0…10.1 × 10^−6^ at 300 K^17,22^) indicate a Δ*α* ≈ (8−9) × 10^−6^ K^−1^ and a resulting stress of 180–200 MPa for Δ*T* = 223 K, which exceeds the tolerable stress of mechanically reliable AFB composites of dissimilar materials^21^ by ~10×. We therefore chose to use undoped YLF instead of sapphire as the thermal link material, nominally yielding Δ*α*≈0 and thus a stress-free adhesive-free bond between the YLF:10%Yb cooling crystal and the undoped YLF thermal link. However, this results in a ~14× lower thermal conductivity of YLF (*κ*_*a*_ = 24 W/m K and *κ*_*c*_ = 34 W/m K at 100 K^16^) than that of sapphire. The undoped YLF thermal link, therefore, must be as short as possible to minimize the temperature difference between the payload and YLF:Yb crystal, while still providing good fluorescence light rejection.

**Fig. 4:**
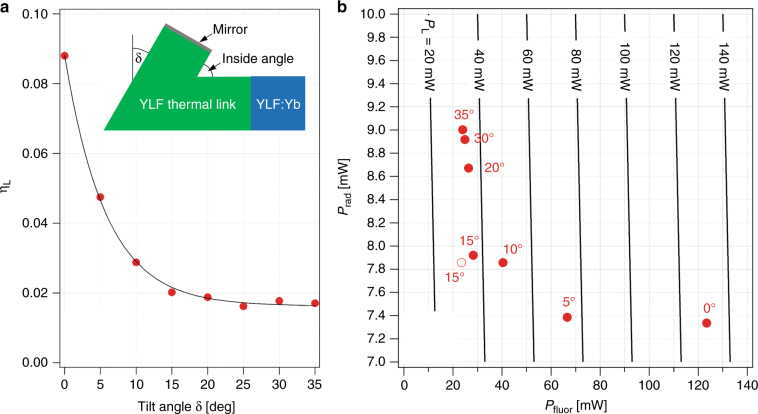
Optimization of the YLF thermal link geometry. **a** The fraction of fluorescence power reaching the link/mirror interface *η*_*L*_ for different tilt angles δ as obtained from a raytracing simulation (red circles) and a fit of the data to a single exponential function (black line). The inset shows a cross-sectional view of the kinked thermal link (green) attached to the YLF:Yb laser-cooling crystal (blue) and coated with a mirror on the interface that attaches to the coldfinger (see Fig. 1). The tilt angle δ is indicated. **b** Calculated heat load on the thermal link from thermal radiation *P*_rad_ vs. the optical heat load due to fluorescence absorption at the link mirror *P*_fluor_ for links with different *δ* values. The calculation assumed link and clamshell temperatures of 135 and 300 K, respectively, *P*_in_=47 W, *η*_cpl_ = 0.999, *η*_ext_ = 0.996, and *R*_*m*_ = 0.97. Link geometries with an inside angle of (90^o^−δ) and 90^o^ are shown as filled and open circles, respectively. Lines of constant total heat load *P*_L_ = *P*_fluor_ + *P*_rad_ are indicated

Given these constraints, we optimized the YLF thermal link in the geometry of a short kinked YLF waveguide (Fig. 4a, inset). The goal was to minimize the total heat load introduced by the thermal link *P*_*L*_ = *P*_fluor_+*P*_rad_, where *P*_fluor_ is the fraction of fluorescence power absorbed at the thermal link mirror surface (see Fig. 1) and *P*_rad_ is the heat load due to radiative heat transport from the coated clamshell surface (warm) to the thermal link (cold). Our measurements have shown that negligible heat is produced by the absorption of the fluorescence in the bulk of the YLF thermal link. We performed an optical raytracing simulation of the light transport in the thermal link using the FRED Optical Engineering software (Photon Engineering, LLC, Tucson, AZ, USA). A 4.1 × 4.1 mm^2^ link cross section was chosen to match the width of the existing YLF:10%Yb crystal. The model used $\bar \lambda _f$= 1004.7 nm as measured for YLF:Yb at 135 K, and a corresponding averaged refractive index of *n* = (2*n*_*o*_ + *n*_*e*_)/3 = 1.456, where *n*_*o*_ = 1.448 and *n*_*e*_ = 1.471^23^, was used for both undoped YLF and YLF:10%Yb. The fluorescence was represented by 10^6^ rays that were uniformly and isotropically emitted within the attached YLF:Yb crystal. As shown in Figure 4a, an exponential decrease of the fraction of fluorescence power reaching the link/mirror interface *η*_*L*_ occurs when increasing the tilt angle *δ*; *η*_*L*_ does not significantly change for *δ* > 15°*.* This simulation yields *P*_fluor_ = *P*_in_*η*_cpl_*η*_ext_*η*_*L*_(1 − *R*_*m*_), where *R*_*m*_ is the reflectance of the mirror, which is set as 0.97 for the sputtered silver layer used in this study, and *P*_in_ = 47 W, *η*_cpl_ = 0.999, and *η*_ext_ = 0.996 are assumed. The radiative heat load on the thermal link is approximately $P_{\mathrm{rad}} = \varepsilon _lA_l\sigma \left( {T_c^4 - T_l^4} \right)/\left( {1 + \chi } \right)$. A 1-mm gap is assumed between the thermal link and the TiNOX-coated clamshell surfaces as well as *ε*_*l*_=0.9 (from CaF_2_^24^), *ε*_*c*_ = 0.05 (TiNOX solar absorber datasheet), *T*_*l*_ = 135 K, and *T*_*c*_ = 300 K. Figure 4b plots *P*_rad_ vs. *P*_fluor_ for thermal links with different *δ*. The *δ* = 15° design achieves the lowest *P*_*L*_, whereas links with a smaller *δ* have a significantly larger *P*_fluor_ due to the greater *η*_*L*_, and links with greater *δ* have a slightly larger *P*_rad_ due to their larger surface areas *A*_*l*_ and *A*_*c*_. The link performance is slightly improved by providing a 90° inside angle (see inset Fig. 4a), a geometry that is also more favorable for fabrication than geometries with smaller inside angles. The final thermal link design (Fig. 2c) is estimated to produce a total heat load of *P*_*L*_ = 31.3 mW, of which 75% is due to residual mirror absorption. Compared with a typical *P*_cool_ = 470 mW (for *P*_in_ = 47 W, *η*_cpl_ ≈ 1, and *η*_*c*_ = 0.01), the heat load introduced by the YLF thermal link is relatively small.

### Silica aerogel supports

The laser-cooled assembly consisting of the YLF:Yb crystal, YLF thermal link, mirror, coldfinger, and payload (Fig. 1) must be mounted within the closely fitting clamshell structure in a manner that introduces minimal thermal conduction from the clamshell to the cooled assembly, is mechanically reliable, and can be easily assembled. Earlier experiments using silica optical fibers with sharpened tips as support elements were difficult to assemble and mechanically unreliable. The new approach implemented in this study uses several silica aerogel cylinders to support the cooled assembly under the coldfinger. Silica aerogels are open-celled mesoporous SiO_2_ networks with low mass densities of typically 0.01–0.2 g/cm^3^ as well as low optical absorption in the visible and near-infrared spectral range. Pure SiO_2_ aerogels have low thermal conductivities of ~0.004 W/m K (at 300 K) and ~0.001 W/m K (at 100 K) in a vacuum^25^, making them attractive for use as support structures in optical cryocoolers. The laser-cooled assembly (19.1 g total weight) rested on three 5 mm diameter × 5 mm tall aerogel cylinders (58.9 mm^2^ total area), thus producing a pressure of 3.18 kPa. Four additional aerogel cylinders were used around the outside perimeter of the coldfinger base to laterally secure the assembly. No mechanical degradation of the aerogel cylinders was observed under these conditions, which is consistent with the compressive strength of silica aerogels, for which values of 1 MPa (0.25 g/cm^3^)^26^ and 0.047–0.11 MPa (0.1 g/cm^3^)^27^ have been reported.

### Laser-cooled assembly and clamshell

**Fig. 5:**
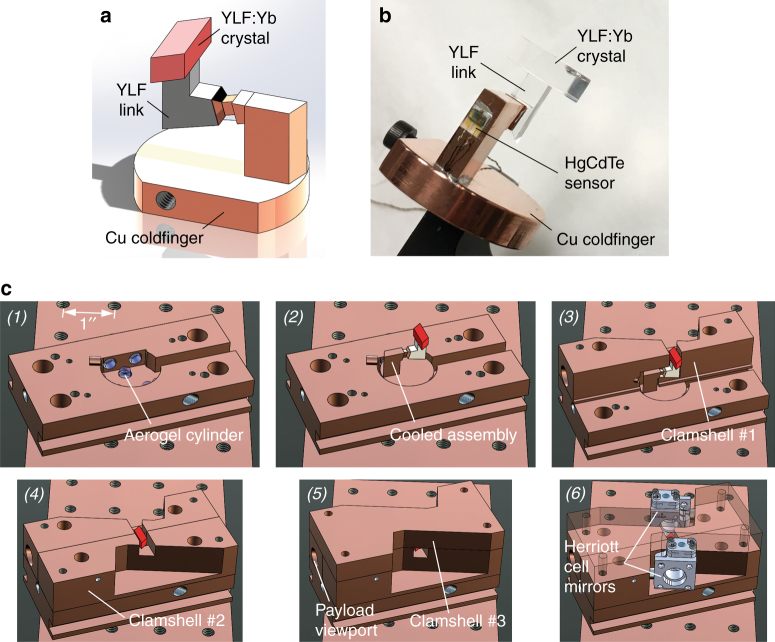
Solid-state optical cryocooler design and assembly. **a** Cooled assembly consisting of a YLF:Yb crystal, YLF thermal link, and copper coldfinger. **b** Image of the cooled assembly showing the HgCdTe sensor payload attached to the coldfinger. **c** Cryocooler assembly sequence consisting of the insertion of seven aerogel cylinders (1), placement of the cooled assembly (2), installation of side and top clamshell layers (3,4,5), and positioning and alignment of Herriott cell mirrors (6)

Earlier solid-state optical cryocooler designs envisioned attaching the payload directly to the coated side of the thermal link^19,28^, an approach that creates significant size constraints for the placement of the support elements and potentially larger payloads. We therefore introduced a copper coldfinger in the present cryocooler, allowing for more space to accommodate larger payloads, aerogel supports, as well as other instrumentation, such as a temperature sensor and heating element. Figure 5a, b show the cooled assembly consisting of the YLF:Yb crystal/YLF thermal link unit and the HgCdTe infrared photo-sensor chip (InfraRed Associates, Inc.) attached to the copper coldfinger. The necked shape of the coldfinger near the thermal link interface was created to provide an optical baffle that prevented residual fluorescence from reaching the payload.

The clamshell serves as the heat sink for the fluorescence emitted by the YLF:Yb crystal and must be designed to minimize the radiative heat loads [Eqs. (2)–(4)] it imparts on the cooled assembly, which requires a geometry that closely envelops the cooled assembly to minimize the surface area and maximize *χ*. Figure 5c shows the assembly sequence of the layered structure, which allows for a stepwise installation of the clamshell at a separation of 1 mm around the cooled assembly. We used a TiNOX Energy solar absorber (Almeco GmbH, Bernburg, Germany) glued to the inside surfaces of the clamshell with silver epoxy to provide high absorbance of the YLF:Yb fluorescence and low thermal emissivity. The clamshell also included a viewport that provided optical access to the HgCdTe sensor payload.

### Laser cooling of a HgCdTe sensor

**Fig. 6:**
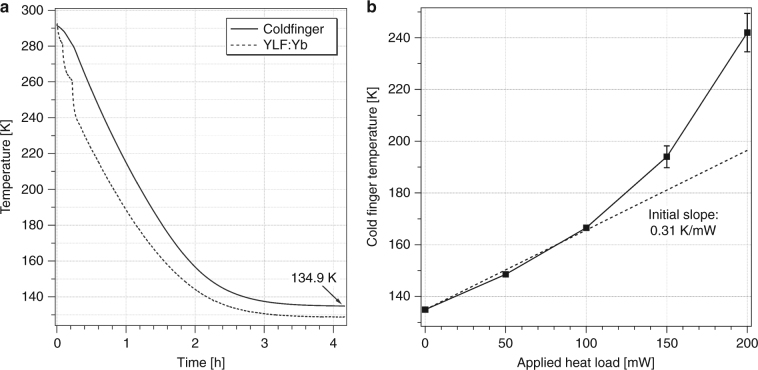
Performance of the solid-state optical cryocooler. **a** Temperature of the coldfinger (solid trace) and YLF:10%Yb crystal (dotted trace) as a function of time after turning on the 1020-nm laser in steps from 0, 12, 25, to 47 W. A steady-state coldfinger temperature of 134.9 K was reached after 4 h. **b** Heat load curve of the cryocooler pumped at 47 W, showing an initial slope of 0.31 K/mW

Figure 6a shows the coldfinger temperature (measured with a silicon diode) and the cooling crystal temperature (measured by DLT) as a function of time after turning on the 1020-nm laser. The 1020 nm laser power was increased in steps from 0, 12, 25, to 47 W as a precautionary measure to reduce the potentially large temperature gradient and associated thermally induced stresses between the YLF:Yb crystal and coldfinger that develop at early times. This is evident in the stepwise decrease of the YLF:Yb crystal temperature as measured by DLT. The coldfinger temperature reached 134.9 K after 4 h, representing the first ever demonstration of cooling a payload to the cryogenic regime using solid-state optical refrigeration. Furthermore, these data experimentally validate the various geometry and material choices for the thermal link, coldfinger, aerogel supports, and clamshell discussed above.

The results shown in Figure 6a were obtained without an electrical current flowing through the HgCdTe sensor, i.e., without an external heat load introduced by the payload. A separate resistor installed in the coldfinger base allowed for the application of well-defined heat loads and measurement of the corresponding increase in the coldfinger temperature. Figure 6b presents the heat load curve obtained with the YLF:10%Yb crystal pumped at 47 W. The initial slope is 0.31 K/mW, a value that is characteristic for this particular cryocooler. Powering up the HgCdTe sensor through a Fourier-Transform Infrared (FTIR) spectrometer (Midac M4401) at the 134.9 K base temperature resulted in a temperature increase of ~2.5 K, which corresponds to an estimated 8 mW heat load introduced by the active HgCdTe sensor. This value is in good agreement with the 8.8 mW heat load calculated from the 22.5 mA bias current and 17.3 Ω resistance (at 134.9 K) of the HgCdTe sensor.

**Fig. 7:**
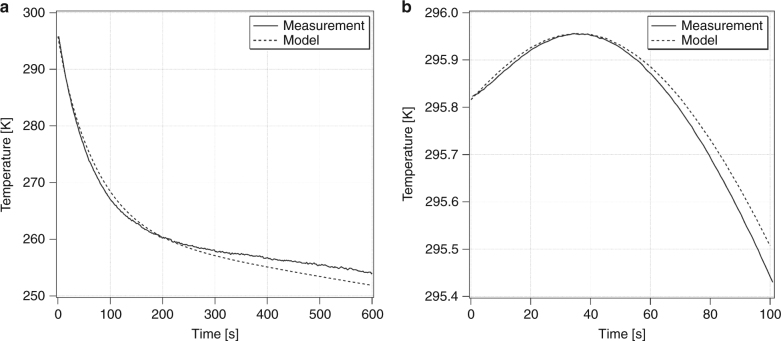
**a** YLF:Yb cooling crystal temperature (*T*_*x*_) and **b** copper coldfinger temperature (*T*_*f*_) at early times. The measured (solid lines) and calculated (dotted lines) temperatures are shown as a function of time after turning on the 1020-nm pump laser at *t* = 0 with *N*_rt_ = 1

The thermal model presented above [Eqs. (2)–(4)] allows us to gain insight into the roles of the various terms in the observed temperature dynamics. The cooling crystal temperature (*T*_*x*_) initially drops rapidly with the slope given by ≈ −*η*_*c*_(*T*_*c*_)*P*_abs_(*T*_*c*_)/*C*_*x*_(*T*_*x*_) before the radiative load (from the chamber) and conductive load (through the link) slow down this process, as shown in Figure 7a. The coldfinger, however, initially experiences a small temperature increase due to the small fluorescence leakage *β*_*l*_ at a rate given by ≈*β*_*l*_*η*_ext_*P*_abs_(*T*_*c*_)/*C*_*f*_(*T*_*c*_) before cooling from the crystal reverses this process in a time scale approximated by *τ* ≈ *K*_*l*_(*T*_*c*_)/*C*_*l*_(*T*_*c*_), as shown in  Figure 7b. Beyond this point, both the crystal and cold-finger continue to cool and reach the steady-state condition with a temperature difference$(T_f - T_x)_{\mathrm{final}} \approx 2P_{\mathrm{load}}^{\mathrm{link}}/K_l(T_l)$, where $P_{load}^{link}$ denotes the total heat load power (parasitic and useful) carried through the link. The data used in Figure 7 and the corresponding fits were obtained for *N*_*rt*_ = 1 (one laser roundtrip). The parameters obtained from these fits are *β*_*l*_≈0.2%, *η*_*c*_(*T*_*c*_)≈1.2% at λ=1020 nm (corresponding to *η*_*ext*_≈0.992), *α*_*b*_≈2.1×10^−4^*cm*^−1^, and ${\it{\epsilon }}_x \approx {\it{\epsilon }}_l \approx 0.38$. The values for *η*_*ext*_ and *α*_*b*_ are in close agreement with previously measured values for this 10% Yb^3+^-doped YLF crystal. *β*_*l*_ also agrees with the calculated value from the raytracing model. *T*_*c*_ was fixed at 283.15 K, which is the final measured temperature of the clamshell. With the above parameters fixed, the model predicts a final coldfinger temperature *T*_*f*_ = 135.0 K with a temperature drop of Δ*T* = *T*_*f*_−*T*_*x*_ = 6.8 K across the link when the number of roundtrips is increased to *N*_rt_≥30, as expected in the Herriott cell arrangement. Considering the simplicity of our model, these calculated values are in excellent agreement with our observed values of *T*_*f*_ = 134.9 K and Δ*T* = 6.1 K (Fig. 6a). The above calculations assumed a negligible heat lift (*P*_lift_ = 0 mW) in comparison to the existing radiative and fluorescence loads. Furthermore, the measured initial slope of 0.31 K/mW (Fig. 6b) is in good agreement with ∂*T*_*f*_/∂*P*_lift_ ≈ 0.31 K/mW obtained from the model calculations.

### FTIR spectroscopy with the laser-cooled HgCdTe sensor

**Fig. 8:**
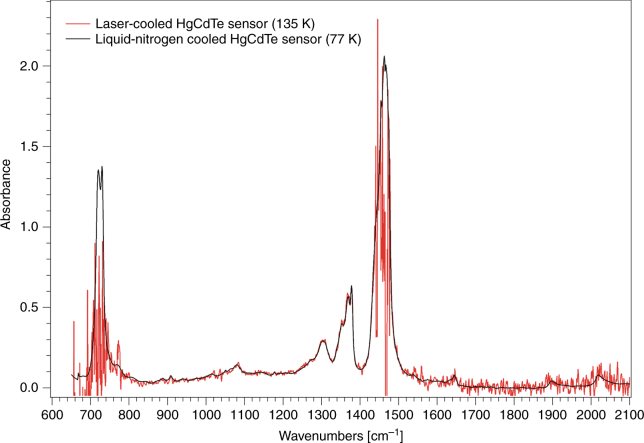
FTIR spectroscopy using a laser-cooled HgCdTe infrared sensor. FTIR spectrum of a sheet of low-density poly-ethylene (LDPE) measured with the laser-cooled HgCdTe sensor (135 K, red trace) and, for comparison, an equivalent liquid-nitrogen cooled HgCdTe sensor (77 K, black trace). The larger relative noise observed at the extremes of the spectra is simply a result of the smaller signal (HgCdTe sensitivity and/or source brightness) in these regions; the noise amplitude is wavelength-independent

In a final experiment, the laser-cooled HgCdTe sensor was used as part of an FTIR spectrometer and the infrared absorption spectrum of a sheet of low-density poly-ethylene (LDPE) was measured (Fig. 8). This is the first demonstration of a sensor cooled by solid-state optical refrigeration used as part of a practical device. Figure 8 shows the absorbance spectrum of the same sample measured with an equivalent HgCdTe sensor cooled with liquid nitrogen to 77 K. The bandgap energy *E*_*g*_ = *hc*/*λ* of this HgCdTe sensor corresponds to a cutoff wavelength of *λ* = 16.7 µm. The primary noise source is due to statistical fluctuations of the thermally activated dark current within the detector element. The dark current *I*_*d*_ is proportional to the number of carriers that are thermally excited across the bandgap and thus varies approximately as *I*_*d*_∝exp(−*E*_*g*_/*k*_*B*_*T*_*f*_),with the corresponding noise amplitude varying as $\sqrt {I_d}$. The detector noise amplitude is therefore expected to be ~11× greater at 135 K than at 77 K, which is consistent with the observed lower signal-to-noise ratio in the measurement using the laser-cooled sensor.

## Conclusions

We used solid-state optical refrigeration to cool a payload to cryogenic temperatures for the first time, which represents a breakthrough in this field and opens the door to using this technology for a variety of applications that benefit from a reliable cryogenic refrigerator without moving parts and associated vibrations. The all-solid-state optical cryocooler was enabled by four key elements: First, a high-quality YLF:Yb crystal with a low *α*_*b*_ and high *η*_ext_ provided efficient cooling at the heart of the cryocooler. Second, the use of a coldfinger provided the design flexibility required to incorporate the sensor payload, support structure, and diagnostic components. Third, the results show that hydrophobic silica aerogels can provide excellent thermal insulation as well as sufficient mechanical strength to support the laser-cooled assembly. Fourth, we demonstrated that a thermo-mechanically reliable van-der-Waals bond can be created between Yb^3+^-doped and undoped YLF, which allowed the incorporation of a transparent thermal link without the use of optical adhesives. These approaches are not without new challenges. The relatively poor thermal conductivity of the YLF thermal link creates a larger than desired temperature gradient between the payload and cooling crystal, which limits the base temperature of the payload. Thermal link materials that have significantly greater thermal conductivity and a CTE that is comparable to the cooling crystal are required to realize the full potential of optical refrigeration. The silica aerogel supports performed well in the present static application. More studies are needed to assess their thermo-mechanical performance under conditions of mechanical shock and vibration.
